# Supplementary material for: Prevalence and risk factors of postpartum depression within one year after birth in urban slums of Dhaka, Bangladesh
Source: PLoS One. 2019 May 2;14(5):e0215735. doi: 10.1371/journal.pone.0215735 (PMC6497249; doi:10.1371/journal.pone.0215735)
Supplement: S2 File — (DOCX) [file pone.0215735.s002.docx]

**Supplementary file 2: Bivariate and Multivariable Regression Model Parameter and Characteristics**

**Table 1: Parameters of bivariate regression model (GLM model with ‘poisson’ family and ‘log’ link)**

| **Indicators** | **e^β*^** | **95%CI^§^** | **Log likelihood** | **Deviance** | **AIC^¥^** | **BIC^€^** |
| --- | --- | --- | --- | --- | --- | --- |
| **Socio-economic factors** |  |  |  |  |  |  |
| Respondent’s age |  |  | -261.3 | 242.7 | 1.57 | -1701.2 |
| 13-19 | 1 |  |  |  |  |  |
| 20-29 | 1.3 | 0.8, 2.0 |  |  |  |  |
| 30-49 | 1.7 | 0.9, 2.6 |  |  |  |  |
| Wealth quintile |  |  | -284.5 | 273.0 | 1.54 | -1926.9 |
| 1st quintile (Comparatively poor) | 1.2 | 0.7, 2.1 |  |  |  |  |
| 2nd quintile | 1.5 | 0.9, 2.5 |  |  |  |  |
| 3rd quintile | 1.0 | 0.6, 1.8 |  |  |  |  |
| 4th quintile | 1.2 | 0.7, 2.0 |  |  |  |  |
| 5th quintile (Comparatively rich) | 1 |  |  |  |  |  |
| Education of respondent |  |  | -283.2 | 270.4 | 1.52 | -1941.4 |
| No education/signed only | 1.8 | 1.1, 3.2 |  |  |  |  |
| Primary | 1.4 | 0.8, 2.4 |  |  |  |  |
| Secondary or higher | 1 |  |  |  |  |  |
| Current profession |  |  | -284.2 | 272.4 | 1.52 | -1945.2 |
| Working | 1.7 | 1.0, 2.7 |  |  |  |  |
| Not working | 1 |  |  |  |  |  |
| Job loss due to pregnancy |  |  | -254.8 | 249.6 | 1.48 | -1768.4 |
| Yes | 1.5 | 1.1, 2.2 |  |  |  |  |
| No | 1 |  |  |  |  |  |
| No of children |  |  | -283.9 | 271.7 | 1.52 | -1939.9 |
| 1 | 1 |  |  |  |  |  |
| 2 | 1.2 | 0.8, 1.7 |  |  |  |  |
| 3+ | 1.5 | 1.0, 2.3 |  |  |  |  |
| **Pregnancy related factors** |  |  |  |  |  |  |
| History of miscarriage or still birth or child death |  |  | -284.3 | 272.7 | 1.52 | -1944.9 |
| Yes | 1.4 | 1.0, 2.0 |  |  |  |  |
| No | 1 |  |  |  |  |  |
| Pregnancy type |  |  | -277.7 | 259.3 | 1.49 | -1958.3 |
| Intended pregnancy | 1 |  |  |  |  |  |
| Unintended pregnancy | 2.0 | 1.4, 2.7 |  |  |  |  |
| Gestational age |  |  | -279.9 | 268.0 | 1.54 | -1887.5 |
| Pre-term birth | 1.3 | 0.8, 1.9 |  |  |  |  |
| Term birth | 1 |  |  |  |  |  |
| Birth weight of last child |  |  | -258.9 | 245.8 | 1.55 | -1697.1 |
| Low birth weight | 1.1 | 0.8, 1.5 |  |  |  |  |
| Normal weight | 1 |  |  |  |  |  |
| Age of the last child |  |  | -258.1 | 274.2 | 1.54 | -1924.7 |
| <3 months | 1 |  |  |  |  |  |
| 3<6 months | 0.9 | 0.6, 1.4 |  |  |  |  |
| 6<9 months | 1.1 | 0.7, 1.7 |  |  |  |  |
| 9<12 months | 1.1 | 0.7, 1.8 |  |  |  |  |
| Neo-natal complications |  |  | -271.2 | 262.4 | 1.52 | -1837.9 |
| Yes | 1.2 | 0.9, 1.8 |  |  |  |  |
| No | 1 |  |  |  |  |  |
| Child delivery cost management by borrowing/sold asset/mortgage |  |  | -282.2 | 268.3 | 1.51 | -1949.3 |
| Yes | 1.6 | 1.2, 2.3 |  |  |  |  |
| No | 1 |  |  |  |  |  |
| Perceived antenatal stress |  |  | -266.4 | 236.8 | 1.43 | -1980.9 |
| Yes | 2.8 | 2.0, 4.0 |  |  |  |  |
| No | 1 |  |  |  |  |  |
| At least one depressive symptom reported to develop during pregnancy |  |  | -261.1 | 226.3 | 1.39 | -1991.4 |
| Yes | 3.6 | 2.4, 5.3 |  |  |  |  |
| No | 1 |  |  |  |  |  |
| **Family support and intimate partner violence** |  |  |  |  |  |  |
| Mother-in-law take care of child and help in household work |  |  | -65.2 | 56.4 | 1.66 | -290.7 |
| Always/Often | 1 |  |  |  |  |  |
| Sometimes/Rarely/ Never | 1.6 | 0.8, 3.2 |  |  |  |  |
| Husband take care of child and help in household work |  |  | -260.0 | 244.1 | 1.55 | -1712.5 |
| Always/Often | 1 |  |  |  |  |  |
| Sometimes /Rarely/Never | 1.4 | 1.0, 1.9 |  |  |  |  |
| Share personal feelings with husband |  |  | -253.2 | 230.4 | 1.51 | -1726.1 |
| Always/Often | 1 |  |  |  |  |  |
| Sometimes/ Rarely/ Never | 2.2 | 1.5, 3.1 |  |  |  |  |
| Intimate partner violence before last pregnancy |  |  | -271.6 | 249.2 | 1.46 | -1961.6 |
| Yes | 3.0 | 1.9, 4.9 |  |  |  |  |
| No | 1 |  |  |  |  |  |
| Intimate partner violence during last pregnancy |  |  |  |  |  |  |
| Yes | 2.3 | 1.6, 3.2 | -273.2 | 252.3 | 1.47 | -1958.4 |
| No | 1 |  |  |  |  |  |
| Intimate partner violence before or during pregnancy period |  |  |  |  |  |  |
| Yes | 3.0 | 1.9, 4.9 | -272.8 | 249.6 | 1.46 | -1968.1 |
| No | 1 |  |  |  |  |  |

*e^β^= Exponential of co-efficient of variables=Prevalence Ratio (PR)

**^§^**95% CI=95% confidence interval

**^¥^**AIC=Akaike information criterion

**^€^**BIC=Bayesian information criterion

**Table 2: Parameters of multivariable regression model (GEE model with ‘poisson’ family and ‘log’ link)**

| **Indicators** | **e^β*^** | **95%CI^§^** | **Tolerance** | **Wald chi-square** | **Prob.>chi square** |
| --- | --- | --- | --- | --- | --- |
| **Full model parameters** |  |  | **2.694e-07** | **71.77** | **<0.0001** |
| Education of respondent |  |  |  |  |  |
| No education/signed only | 1.2 | 0.7, 2.1 |  |  |  |
| Primary | 0.9 | 0.5, 1.6 |  |  |  |
| Secondary or higher | 1 | 1 |  |  |  |
| Current profession |  |  |  |  |  |
| Working | 1.9 | 1.1, 3.3 |  |  |  |
| Not working | 1 | 1 |  |  |  |
| Job loss due to pregnancy |  |  |  |  |  |
| Yes | 1.5 | 1.0, 2.1 |  |  |  |
| No | 1 | 1 |  |  |  |
| No of children |  |  |  |  |  |
| 1 | 1 | 1 |  |  |  |
| 2 | 1.0 | 0.7, 1.5 |  |  |  |
| 3+ | 1.0 | 0.6, 1.5 |  |  |  |
| History of miscarriage or still birth or child death |  |  |  |  |  |
| Yes | 1.4 | 1.0, 2.0 |  |  |  |
| No | 1 | 1 |  |  |  |
| Pregnancy type |  |  |  |  |  |
| Intended pregnancy | 1 | 1 |  |  |  |
| Unintended pregnancy | 1.8 | 1.3, 2.5 |  |  |  |
| Child delivery cost management by borrowing/sold asset/mortgage |  |  |  |  |  |
| Yes | 1.3 | 0.9, 1.9 |  |  |  |
| No | 1 | 1 |  |  |  |
| At least one depressive symptom reported to develop during pregnancy |  |  |  |  |  |
| Yes | 2.5 | 1.7, 3.8 |  |  |  |
| No | 1 | 1 |  |  |  |
| Intimate partner violence before or during pregnancy period |  |  |  |  |  |
| Yes | 2.0 | 1.2, 3.3 |  |  |  |
| No | 1 | 1 |  |  |  |

*e^β^= exponential of co-efficient of variables= Adjusted Prevalence Ratio (APR), estimates adjusted for clustering at slum level and wealth score.

**^§^**95% CI=95% confidence interval
